# Successful Same-Cycle Blastocyst Transfer following Laparoscopic Ovarian Detorsion: A Report of Two Cases and Literature Review

**DOI:** 10.1155/2014/806378

**Published:** 2014-04-29

**Authors:** Mohamad Irani, Reshef Tal, David B. Seifer, Richard V. Grazi

**Affiliations:** ^1^Department of Obstetrics and Gynecology, Maimonides Medical Center, 4802 Tenth Avenue, Brooklyn, NY 11219, USA; ^2^Division of Reproductive Endocrinology and Infertility, Maimonides Medical Center, Brooklyn, NY, USA

## Abstract

Ovarian stimulation increases the risk of ovarian torsion. During an in vitro fertilization (IVF) cycle, the effects of ovarian torsion on retrieved oocytes and subsequent pregnancy chances are not clear. Moreover, no cases of ovarian torsion occurring following oocyte retrieval but prior to same-cycle embryo transfer have been reported. Such cases present a clinical dilemma with respect to optimal timing of embryo transfer. We report two cases of a 41-year-old and a 32-year-old infertility patients undergoing IVF who were diagnosed with ovarian torsion within several days following oocyte retrieval. Both patients were treated by early laparoscopic evaluation and detorsion followed by day five embryo transfer, resulting in successful pregnancies. Therefore, after prompt laparoscopic ovarian untwisting of a torsed ovary following egg retrieval, embryo transfer may be performed as originally scheduled during the concurrent cycle leading to favorable pregnancy outcomes.

## 1. Introduction

Ovarian torsion occurs when the ovary makes a partial or complete rotation on its ligamentous supports, often resulting in compromise of its blood supply. It accounts for 3% of gynecological emergencies [1]. Ovarian mass, pregnancy, and ovarian stimulation are risk factors for ovarian torsion [1]. Prompt diagnosis and treatment are crucial for preservation of the ovary. However, clinical presentation of ovarian torsion is usually vague and includes nonspecific signs and symptoms, often presenting a diagnostic challenge.

Bilateral ovarian enlargement after ovulation induction increases the risk of ovarian torsion [2]. The incidence of ovarian torsion in in vitro fertilization (IVF) cycles is approximately 0.1% [3]. Most ovarian torsions occur after embryo transfer, usually in the setting of ovarian hyperstimulation syndrome [4]. Laparoscopic unwinding of a torsed ovary diagnosed after embryo transfer can preserve ovarian function without adversely affecting pregnancy [5]. Successful laparoscopic management of ovarian torsion during an IVF cycle prior to oocyte retrieval has also been reported. However, the effects of ovarian torsion on the retrieved oocytes and subsequent pregnancy chances are not clear [4, 6]. Moreover, no cases of ovarian torsion following oocyte retrieval but prior to embryo transfer have been reported, and they present a clinical dilemma with respect to embryo transfer timing.

We report two cases of ovarian torsion that occurred one day and three days after egg retrieval and were appropriately diagnosed and treated by laparoscopic detorsion, followed by embryo transfer on day five of the same IVF cycle. To our knowledge, we report the first two cases of ovarian torsion after oocyte retrieval resulting in successful pregnancies following same-cycle day five embryo transfer.

## 2. Case 1

A 41-year-old woman presented in 2012 to our office with history of secondary infertility for the past year. The patient and her partner were counseled for intracytoplasmic sperm injection (ICSI) due to combined male and tubal factor infertility. The patient underwent stimulation using a combination of 225 IU/day recombinant follicle stimulating hormone with gonadotropin-releasing hormone antagonist. Thirty-six hours following human chorionic gonadotropin (hCG) injection, 12 oocytes were retrieved, 11 oocytes were inseminated, and 8 were fertilized.

**Figure 1:**
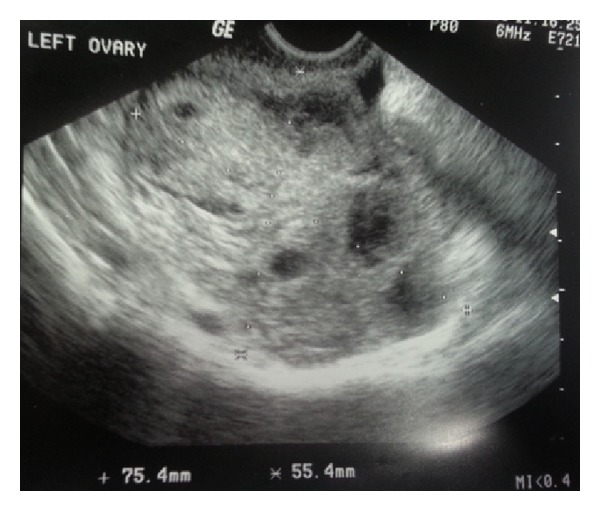
Left ovary measuring 75.4 mm × 55.4 mm.

One day after egg retrieval, the patient presented to our office complaining of severe left lower quadrant pain, associated with nausea and vomiting. Her vital signs were stable and abdominal exam revealed mild left lower quadrant tenderness with no rebound or guarding. Transvaginal sonogram (Figure 1) showed left ovary measuring 75 × 55 mm, right ovary measuring 71 × 43 mm, mild free fluid, and normal Doppler blood flow to both ovaries. Her white blood cell count was 20.4 × 10^3^ cells/*μ*L with 90% neutrophils. Due to high suspicion of left ovarian torsion, the patient was transferred immediately to the hospital for diagnostic laparoscopy. At laparoscopy, left ovarian torsion with multiple small cysts was noted. The left ovary was found to be twisted 180° around the infundibulopelvic ligament and had a viable pinkish color. The left ovary was untwisted successfully and three small cysts were drained. The patient tolerated the procedure well and the postoperative recovery was uneventful.

The patient was informed about the unknown impact of ovarian torsion and detorsion on the implantation rate and pregnancy outcome as well as the absence of evidence supporting embryo cryopreservation versus fresh embryo transfer. She opted to proceed with fresh embryo transfer. On day five following egg retrieval, four blastocysts were transferred under transabdominal ultrasound guidance. Subsequent sonogram four weeks later confirmed a viable six-week intrauterine singleton gestation. She had a vaginal delivery at 41-week gestational age after induction of labor.

## 3. Case 2

A 32-year-old woman presented in 2010 to our clinic with history of primary infertility for two years. She had polycystic ovarian syndrome (PCOS) according to the Rotterdam criteria [7] based on her history of irregular periods and sonographic polycystic ovarian morphology. The patient was initially given 100 mg Clomiphene citrate for ovulation induction followed by intrauterine insemination. After four failed cycles of ovulation induction/intrauterine insemination, a decision was made to proceed with IVF.

**Figure 2:**
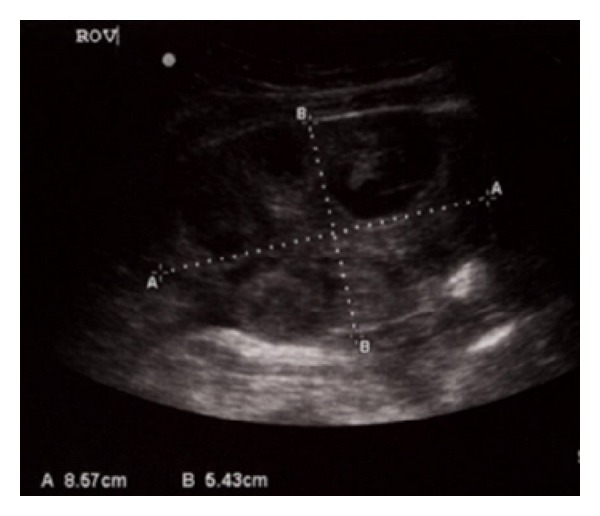
Right ovary measuring 85.7 mm × 54.3 mm.

The patient underwent stimulation using a combination of 75 IU/day recombinant follicle stimulating hormone with gonadotropin-releasing hormone antagonist. Transvaginal retrieval of 22 oocytes was performed 36 hours after hCG administration (5,000 IU), and 17 oocytes were fertilized. Three days after egg retrieval, the patient presented with acute severe right lower quadrant pain associated with nausea and vomiting. Upon presentation to our hospital emergency department, her vital signs were stable and her abdominal exam demonstrated right lower quadrant tenderness and guarding with no rebound tenderness. Pelvic sonogram (Figure 2) revealed minimal free fluid in the cul-de-sac, right ovary measuring 85 × 54 mm, and left ovary measuring 71 × 53 mm with multiple residual follicles. Doppler ultrasound study was not available at the time of evaluation. Her white blood cell count was 14.4 × 10^3^ cells/*μ*L with 89% neutrophils. An emergent laparoscopy revealed a 10 × 12 cm purple right ovary twisted 180° around the right infundibulopelvic ligament in addition to a mild right hydrosalpinx. The left tube and ovary appeared normal. The right ovary was untwisted and regained its pinkish color within three minutes, consistent with a viable ovary. The patient had an uneventful recovery and was discharged home in stable condition on the same day.

The patient was counseled similarly to the patient described in “Case 1.” She also preferred to proceed with fresh embryo transfer. Two blastocysts were transferred on day five under transabdominal ultrasound guidance. Four weeks later, sonogram showed a dichorionic-diamniotic twin gestation of six-week size. She subsequently had a vaginal delivery of a healthy boy and girl at 35 weeks of gestation.

## 4. Discussion

10–20% of ovarian torsion cases occur during pregnancy [1]. Ovarian stimulation, especially when it is associated with ovarian hyperstimulation syndrome, predisposes the ovaries to torsion [8]. Pelvic sonogram is the initial imaging modality for evaluation of ovarian torsion [9]. Doppler studies have high specificity but low sensitivity and may be normal in 60% of torsion cases [9]. Our first case of ovarian torsion had normal Doppler flow before the laparoscopy. This is important because it may lead to false reassurance and delay in the diagnosis and intervention. This delay increases the risk of ovarian necrosis and other sequelae. Laparoscopy is considered the procedure of choice for ovarian detorsion [10]. Long-term follow-up of patients after laparoscopic detorsion performed in the setting of ischemic and nonviable appearance of the torsed ovary revealed that most ovaries regain normal appearance and function [11].

To our knowledge, this is the first report in the literature of cases of ovarian torsion occurring after oocyte retrieval but before embryo transfer, which were followed by detorsion and embryo transfer in the same IVF cycle, resulting in successful pregnancies. Previously, management of ovarian torsion related to IVF cycle by laparoscopic detorsion has been reported with torsion occurring either prior to oocyte retrieval or following embryo transfer.

Surgery in pregnancy is considered to carry potential risks to both mother and fetus, including maternal complications, fetal loss, and preterm birth [12]. However, accumulating evidence suggests that laparoscopic treatment of adnexal masses in the first trimester of pregnancy is safe and effective [13]. Moreover, Mashiach et al. [8] reviewed 154 patients who were hospitalized for OHSS after gonadotropin treatment cycles. Of the pregnant patients, 16% developed ovarian or adnexal torsion. Eleven of 12 pregnant patients had their torsed adnexa conserved during surgery and the outcome of pregnancy was mostly favorable.

Reproductive endocrinologists are usually hesitant to transfer embryos a few days after a stressful event and surgical procedure such as laparoscopic ovarian detorsion due to its unknown impact on implantation rate and pregnancy outcome. However, there is no evidence that laparoscopic ovarian detorsion performed after oocyte retrieval but several days before embryo transfer may adversely affect implantation rate or increase early pregnancy loss.

Robson and Kerin [4] reported a case of ovarian torsion that occurred a few hours prior to egg retrieval. Laparoscopy was performed two hours after egg retrieval demonstrating ovarian torsion, which was successfully treated by detorsion. Embryo transfer was performed on day three after retrieval but the patient did not become pregnant [4]. In another case of ovarian torsion occurring during an IVF cycle prior to oocyte retrieval, Smith et al. [6] reported a successful treatment by laparoscopic detorsion followed by egg retrieval on postoperative day one. Although the fertilization rate of oocytes retrieved from the detorsed ovary was only 40% (compared to 93% from oocytes of the normal ovary), development of the embryos originating from the detorsed ovary was excellent. However, embryo transfer was not performed due to abnormal preimplantation genetic screening results in all embryos [6]. Remarkably, in both cases there were similarly markedly reduced fertilization rates of oocytes from the previously torsed ovary as compared with the contralateral normal ovary, suggesting adverse effects of decreased ovarian arterial flow on oocyte function [4, 6].

Ovarian torsion occurring two days and one week after embryo transfer was reported and was managed by laparoscopic unwinding of the torsed ovary [5, 14]. The interventions resulted in preservation of ovarian function without adverse effects on the pregnancy [5, 14]. Similarly, in our two cases laparoscopic detorsion was able to preserve ovarian function and was followed by successful pregnancies. The results of these cases suggest that ovarian torsion occurring after oocyte retrieval, and its timely laparoscopic intervention, does not have detrimental effects on implantation or early pregnancy outcome.

In summary, these two cases suggest that, after laparoscopic ovarian detorsion of a torsed ovary that occurs following oocyte retrieval, an embryo transfer should not be canceled. Consideration can be given to same-cycle fresh embryo transfer with favorable outcome, thus avoiding cryopreservation of all embryos and delayed frozen embryo transfer.
